# Rationally Designed Hierarchically Structured Tungsten Nitride and Nitrogen‐Rich Graphene‐Like Carbon Nanocomposite as Efficient Hydrogen Evolution Electrocatalyst

**DOI:** 10.1002/advs.201700603

**Published:** 2017-12-08

**Authors:** Yanping Zhu, Gao Chen, Yijun Zhong, Wei Zhou, Zongping Shao

**Affiliations:** ^1^ Jiangsu National Synergetic Innovation Center for Advanced Materials (SICAM) State Key Laboratory of Materials‐Oriented Chemical Engineering College of Chemical Engineering Nanjing Tech University No. 5 Xin Mofan Road Nanjing 210009 P. R. China; ^2^ Department of Chemical Engineering Curtin University Perth Western Australia 6845 Australia; ^3^ College of Energy Nanjing Tech University No. 5 Xin Mofan Road Nanjing 210009 P. R. China

**Keywords:** electrocatalysis, hydrogen evolution reaction, ion‐exchange, tungsten nitride, water splitting

## Abstract

Practical application of hydrogen production from water splitting relies strongly on the development of low‐cost and high‐performance electrocatalysts for hydrogen evolution reaction (HER). The previous researches mainly focused on transition metal nitrides as HER catalysts due to their electrical conductivity and corrosion stability under acidic electrolyte, while tungsten nitrides have reported poorer activity for HER. Here the activity of tungsten nitride is optimized through rational design of a tungsten nitride–carbon composite. More specifically, tungsten nitride (WN*_x_*) coupled with nitrogen‐rich porous graphene‐like carbon is prepared through a low‐cost ion‐exchange/molten‐salt strategy. Benefiting from the nanostructured WN*_x_*, the highly porous structure and rich nitrogen dopant (9.5 at%) of the carbon phase with high percentage of pyridinic‐N (54.3%), and more importantly, their synergistic effect, the composite catalyst displays remarkably high catalytic activity while maintaining good stability. This work highlights a powerful way to design more efficient metal–carbon composites catalysts for HER.

Hydrogen, as a renewable energy material with zero‐emission feature, has been hailed as an ideal sustainable alternative to finite fossil fuels, while one of the key issues to realize the practical use of hydrogen is to find an effective way for its large‐scale generation at low cost. Nowadays, electrochemical water splitting for hydrogen has received tremendous attention considering its high theoretical efficiency, easy accessibility of water, and potentially high hydrogen production rate.^1^, ^2^ Water electrolysis involves oxygen evolution reaction over the anode and hydrogen evolution reaction (HER) over the cathode. The practical energy conversion efficiency, however, strongly relies on the catalytic activity of both electrodes toward the respective reaction. Until now, platinum (Pt) group precious metals are still the most efficient catalysts for HER, but their high cost and scarcity seriously hamper their use in real devices.^3^, ^4^, ^5^ Innovative Pt‐free HER electrocatalysts are urgently needed for realizing large‐scale hydrogen generation from water electrolysis.

The performance of a HER catalyst is closely related to its surface property, electronic conductivity, and number of active sites. To achieve high HER activity, hydrogen adsorption on the catalyst surface should not be too strong or too weak, while the electronic structure of a catalyst can affect the Gibbs adsorption energy of hydrogen over the catalyst surface.^6^ On the other hand, electrocatalytic efficiency is also affected by the electronic conductivity of the catalyst, while the insufficient electron conductivity may cause the formation of Schottky barrier at both catalyst–electrolyte and catalyst–support electrode interfaces, requiring an extra overpotential to overcome, thus leading to decreased energy conversion efficiency.^7^ In addition, the HER performances of the electrocatalysts are also affected by the dispersity of active sites.^7^ Therefore, the design of a good catalyst should take into account of all these three factors.

Many strategies have been applied in the development of precious metal‐free catalysts for HER. Among them, the formation of transition metal with nonmetal element(s), such as C, N, P, S, and B, has turned out to be an attractive way to design new active HER eletrocatalysts.^8^, ^9^, ^10^ Tungsten‐based materials are an important family of catalysts. Alloying W with C, S, P, and N can form active catalysts for hydrodesulfurization (HDS), solar cells, oxygen reduction reaction, and HER.^11^, ^12^, ^13^, ^14^ In particular, in the HER field, tungsten phosphides and tungsten sulfides have emerged as electrocatalysts, but less attention has been paid to the tungsten nitrides.^6^, ^15^ However, the mechanistic commonality of HDS with HER implies that the WN should be potential catalysts for HER, and the similar electronic structure of W with Mo also supports this speculation.^8^ Recently, WN nanorods on carbon cloth has been developed as an HER catalyst in acidic electrolyte, but with relatively low activity.^14^ A further improvement of catalytic activity is still needed to make WN competitive with Pt‐based electrodes. Heteroatoms (N, S, P, or B), in particular N‐doped carbon nanostructures are also widely investigated as potential Pt‐free electrocatalysts for HER. Both experiments and density functional theory (DFT) calculations have confirmed that nitrogen doping can improve the conductivity of the carbon, and the carbon atoms adjacent to N dopants can act as active sites and increase the H* adsorption sites, thus enhancing the HER activity.^16^, ^17^, ^18^ Although featuring tunable molecular structure, superior conductivity, and interesting composition chemistry, the carbon‐based catalysts still suffer from insufficient catalytic activity for HER compared with Pt counterparts.

Coupling transition metal sulphides, carbides, phosphides, or nitrides with carbon materials is found rather effective to create a synergistic effect between the components. Such coupling can enhance dispersity of the metal phase (increased active sites), modulate the electron density as well as the distribution of electronic potential in the hybrid materials (improved hydrogen surface adsorption behavior), and increase the electronic conductivity (enhanced charge transfer process), thus enhancing the HER activity and stability with respect to their individual component.^7^, ^8^ Since the synergy between the two components is highly dependent on the microstructure of the composite, in order to maximize such synergistic effect, the composite should be rationally designed, while the facile synthesis of such composite with maximized synergistic effect is a big challenge.

In this study, a nanocomposite of tungsten nitride (WN*_x_*) nanoparticles decorated on nitrogen‐rich porous graphene‐like carbon nanosheets (WN*_x_*‐NRPGC) as a new hydrogen evolution electrocatalyst is reported, demonstrating remarkable electrocatalytic activity toward HER in acidic electrolyte, comparable or even overperforming other transition metal nitrides. We further propose a facile one‐pot synthesis technique based on ion exchange of resin and molten salt heating under NH_3_ atmosphere for the preparation of such strongly coupled composite. During the one‐step calcination, transformation of the resin skeleton to porous graphene‐like carbon nanosheets, the doping of abundant nitrogen atoms into the carbon structure with preferable pyridinic nitrogen, the formation of WN*_x_* nanoparticles and the uniform anchoring of such WN*_x_* nanoparticles over the surface of carbon nanosheets were realized simultaneously, resulting in the formation of hierarchical porous WN*_x_*‐NRPGC 3D networks. Such hybrid demonstrates outstanding activity and stability, superior to most of the reported non‐Pt HER electrocatalysts in acidic medium to date.

The procedure for one‐pot synthesis of WN*_x_*‐NRPGC hybrid is illustrated as schematically illustrated in **Scheme 1**: An appropriate amount of pretreated D201 was firstly immersed in a sodium tungstate dihydrate (Na_2_WO_4_·2H_2_O) aqueous solution for sufficient time to allow the full exchange of the OH^−^ groups in D201 with WO_4_^2−^ anions. The WO_4_^2−^‐exchanged resin was then thoroughly mixed with KCl/NaCl in powder form and calcined under a flowing NH_3_ atmosphere at elevated temperature for certain time. Upon heating, KCl/NaCl was melted to form a molten salt, which performed both the functions of pore former to generate rich pores in the as‐derived functional carbon and promoter to rearrange the polymer skeleton of resin with sp^2^ hybridized carbons into a 2D graphene‐like nanosheets with sp^3^ hybridized carbons.^19^ Simultaneously, the conversion of WO_4_^2−^ to WN_x_ and the doping of nitrogen into the carbon from both the quaternary amine of resin and the annealing atmosphere of ammonia also appeared. Finally, the 3D‐networked WN*_x_*‐NRPGC was successfully obtained by removal of the porogen in the sample through water washing.

**Scheme 1:**
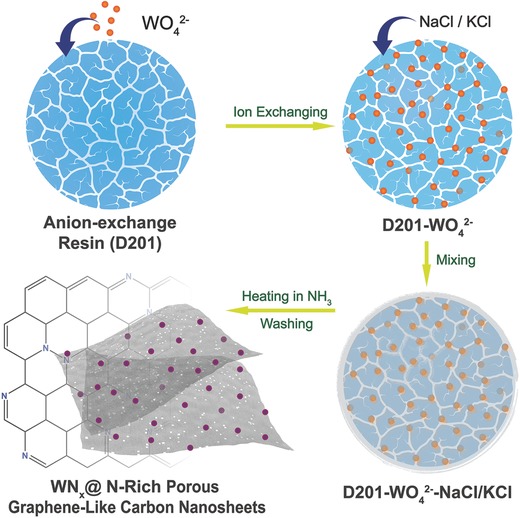
Procedure for the synthesis of the WN*_x_*‐NRPGC composite.

The successful formation of WN*_x_* crystalline phases after the one‐step calcination of the WO_4_^2−^‐exchanged D201 resin was first confirmed by room‐temperature X‐ray diffraction (XRD). As revealed in **Figure** **1**a, all the diffraction peaks of the as‐prepared WN*_x_*‐NRPGC sample are readily indexed based on a mixture of the hexagonal W_4.6_N_4_ (JCPDS no. 77–2001) and the cubic WN (JCPDS no. 75–1012) crystalline phases, while no impurities such as tungsten oxides or tungsten carbides are detected. No obvious diffraction peaks for crystallized carbon phase were detected, suggesting the amorphous nature of the as‐formed carbon, and any weak diffraction peaks for graphitic carbon were masked by the peaks of WN*_x_*. Figure 1b,c shows the particulate morphology of the as‐prepared WN*_x_*‐NRPGC sample, which was composed of graphene‐like nanosheets with their surface decorated with many nanoparticles, and such nanosheets were curled and randomly connected to form a networked architecture. The corresponding transmission electron microscopy (TEM) image as shown in Figure 1d,e demonstrates ≈100 nm in size for the nanoparticles, while the substrate is predominantly in thin lamellar architecture. The high‐resolution TEM (HRTEM) image in Figure 1f exhibits clear lattice fringes with interplanar spacings of 0.22, 0.25, and 0.28 nm for the nanoparticles, corresponding to the (103) and (101) planes of WN and (110) plane of W_4.6_N_4_, respectively. These results confirm the WN nature of the nanoparticles. No crystal lattice for the graphene‐like nanosheets were observed by the HRTEM (Figure S1, Supporting Information), suggesting their amorphous nature, which are then assigned to the amorphous carbon converted from the resin. It suggests, after the one‐step calcination, a hierarchical architecture with nanosized WN*_x_* decorated on graphene‐like amorphous carbon nanosheets was formed. From the electrochemical point of view, such networked architecture ensures efficient electron diffusion, and the nanosized structure of WN*_x_* can bring large numbers of active sites, all of these features benefit the HER.^8^, ^20^

**Figure 1:**
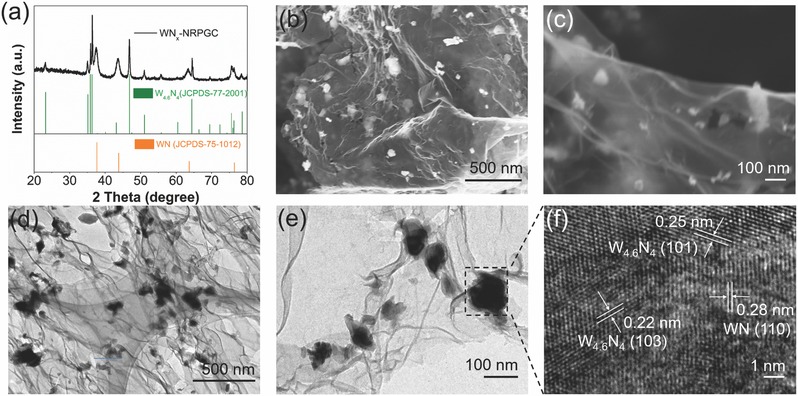
a) XRD pattern, b,c) SEM, d,e) TEM, and f) HRTEM images of the WN*_x_*‐NRPGC composite.

To emphasize the important role of molten‐salt heating for the formation of such graphene‐like carbon nanocomposites, two reference samples were also synthesized for comparison: a nanohybrid of WN*_x_* embedded in the N‐rich carbon (denoted as WN*_x_*‐NRC) through a similar preparation procedure without the introduction of KCl/NaCl during the pyrolysis, and WN*_x_*‐free sample (defined as NRPGC) by pyrolysis of D201 alone in the molten salt. XRD patterns of the reference samples and their corresponding scanning electron microscopy (SEM) images are displayed in Figure S2 (Supporting Information). The diffraction peaks of Figure S2a of the Supporting Information are unambiguously indexed to a mixture of W_4.6_N_4_ and WN, and the corresponding SEM image (Figure S2b, Supporting Information) showed particles of 200–300 nm that were seriously aggregated. According to our previous study, such grains are actually composed of many nanoparticles embedded in amorphous carbon matrix.^21^ As for the WN*_x_*‐free sample (NRPGC), two broad peaks at 2θ = 24.6° and 43.5° appeared in its XRD pattern (Figure S2c, Supporting Information), suggesting its poor graphitization degree in nature.^17^ According to the corresponding SEM image in Figure S2d of the Supporting information, the NRPGC sample had a similar graphene‐like particulate morphology to that of the WN*_x_*‐NRPGC composite. These results suggest that the molten salt played a critical role in the formation of such porous graphene‐like nanosheets from D201, while the tungsten did not. It is well known that lattice defects of a carbon material play an important role in the catalytic activity for HER.^8^, ^18^ To analyze the effect of molten salt heating on the lattice defect in the carbon structure, Raman result of the WN*_x_*‐NRPGC sample was analyzed and compared with those of the WN*_x_*‐NRC and NRPGC samples. According to Figure S3 of the Supporting Information, all three samples showed two prominent peaks at 1343 and 1597 cm^−1^, defined as D and G band, respectively.^8^, ^18^ The intensity ratio of D band to G band is widely accepted as the degree of graphitization, defects, or the domain size of graphitization. It is observed that the *I*_D_/*I*_G_ values for the three samples are similar, (1.30, 1.27, and 1.26, respectively for WN*_x_*‐NRPGC, WN*_x_*‐NRC, and NRPGC). It suggests that both the exchanged WO_4_^2−^ anion and the molten salt actually had negligible impact on the graphitization/defect formation of the carbon from the pyrolysis of resin. X‐ray photoelectron spectroscopy (XPS) measurements were performed to get information about the oxidation state and surface composition of the as‐synthesized WN*_x_*‐NRPGC composite, which is also well connected with the HER activity. The survey spectrum in Figure S4a of the Supporting Information demonstrates the concomitance of W, N, C, and O elements in the composite. **Figure** **2**a shows the high‐resolution core level spectrum of W 4f, which can be fitted to two pairs of peaks for W—N bonds (34.7 and 32.6 eV) in the tungsten nitride and W—O bonds (37.6 and 35.4 eV). The W—O bonds were likely resulted from the inevitable surface oxidation of WN*_x_* nanoparticles upon exposure to air.^22^, ^23^ In the N 1s spectrum (Figure 2b), the individual peak at 397.4 eV is ascribed to the N in WN*_x_* nanoparticles, while the other peaks represent respectively pyridinic (398.8 eV), pyrrolic (400.3 eV), and graphitic (401.7 eV) nitrogen atoms, suggesting the successful incorporation of nitrogen atoms into the carbon structure during the calcination.^8^, ^24^ The salient C–N peak at 285.8 eV of the C 1s spectrum in Figure S4b of the Supporting Information further supports the successful doping of N atoms into carbon structure. The N content in the NRPGC was estimated to as high as 9.5 at% from XPS, which is much larger than those yielded from the commonly used nitrogen source such as melamine, dicyandiamide, and polyaniline.^7^, ^20^, ^25^ The rich nitrogen in the carbon skeleton is expected to further ameliorate the catalytic behavior.

**Figure 2:**
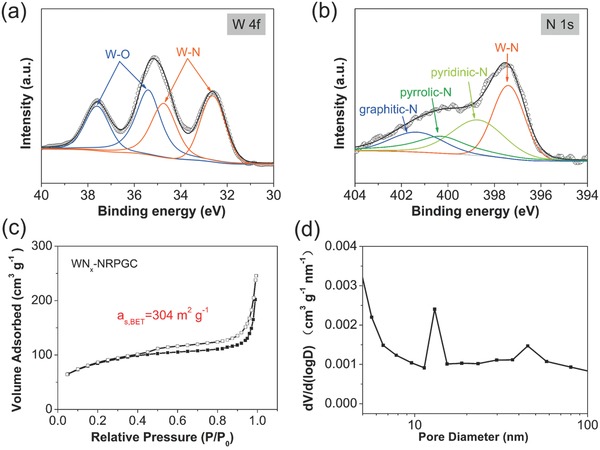
High‐resolution XPS spectra of a) W 4f and b) N 1s of WN*_x_*‐NRPGC. c) N_2_ adsorption–desorption isotherms and d) the corresponding pore size distribution curve of WN*_x_*‐NRPGC.

The microstructure of the as‐prepared WN*_x_*‐NRPGC composite was analyzed by the nitrogen adsorption–desorption measurement. As plotted in Figure 2c, the sample demonstrated a typical IV isotherm with a large hysteresis loop at *P*/*P*_0_ > 0.5, indicating its rich mesopores in nature.^26^, ^27^ The corresponding pore size was further analyzed based on the isotherm, suggesting a hierarchical size distribution centered at 15 nm. According to the Brunauer–Emmett–Teller and Barrett–Joyner–Halenda methods, the WN*_x_*‐NRPGC composite possesses a high specific surface area (SSA) of 304 m^2^ g^−1^ and a large pore volume of 0.35 cm^3^ g^−1^. Such microstructure with large surface area and abundant porosity is expected to allow easy penetration of liquid electrolyte and provide a large number of exposed active sites as an HER electrocatalyst.^8^, ^24^ By contrast, according to the nitrogen adsorption/desorption isotherms (Figure S5a,b, Supporting Information), the WN*_x_*‐NRC sample is mesopore‐free in nature with a quite low SSA value of 25.4 m^2^ g^−1^ and small pore volume of 0.11 cm^3^ g^−1^. Meanwhile, the NRPGC sample also demonstrated rich mesopores (Figure S5c, Supporting Information) and a continuous pore distribution in the range of 6–30 nm (Figure S5d, Supporting Information). Above results suggest that the introduction of molten salt during the synthesis effectively facilitated the formation of a porous structure with rich mesopores. The random stacking of the curled graphene‐like carbons as well as the molten‐salt templating may account for the rich mesopores in the WN*_x_*‐NRPGC composite.

The electrocatalytic performance of the WN*_x_*‐NRPGC composite as an electrode for HER was evaluated in 0.5 m H_2_SO_4_ with a convenient three‐electrode cell with the catalyst loading of 0.362 mg cm^−2^. To determine the optimal synthesis conditions for WN*_x_*‐NRPGC, various samples that were synthesized from different amounts of exchanged W source (Figure S6a, Supporting Information) and annealed at different temperatures (Figure S6b, Supporting Information) were studied by linear sweep voltammetry (LSV) measurements. A concentration of WO_4_^2−^ of 0.3 m and a calcination temperature of 800 °C were determined to result in the best HER activity, which were then selected for the preparation of all the WN*_x_*‐NRPGC composites for following investigations. For comparison, the 20% Pt/C, WN*_x_*‐NRC, bulk WN and NRPGC electrodes were also assessed under the same condition. Here bulk phase WN was synthesized by annealing WO_3_ under ammonia atmosphere at 800 °C for 2 h. The XRD pattern (Figure S7a, Supporting Information) and SEM image (Figure S7b, Supporting Information) confirm the formation of WN phase with large grains.

**Figure** **3**a displays the LSV curves on the reversible hydrogen electrode scale of the various working electrodes (after IR‐drop corrections). As expected, the Pt/C electrode is highly active toward HER, demonstrating comparable activity to that as reported in literature.^25^, ^28^ As expected, the bulk‐phase WN electrode showed rather poor HER activity, as indicated by a large initial overpotential (*U*_onset_) of 331 mV and large overpotential of 492 mV at the representative current density of 10 mA cm^−2^ (η_10_), respectively, which can be explained by its low number of active sites, poor electronic conductivity, and nonoptimized surface properties. As compared to the bulk‐phase WN, the WN*_x_*‐NRC sample exhibited dramatically enhanced electrocatalytic activity, reflected by the decreased *U*_onset_ value of 123 mV and a reduced overpotential of 255 mV at 10 mA cm^−2^. Clearly, the increased active sites due to the reduced particle size of WN*_x_* phase should account at least in part to such improvement. By applying molten‐salt heating during the synthesis, the as‐derived WN*_x_*‐NRPGC catalyst showed further improved HER performance, and the catalyst needs only 132 mV to drive 10 mA cm^−2^ (Table S2, Supporting Information), which is favorably compared to most of the reported precious metal‐free electrocatalysts in acidic solutions, including W_2_C/MWNT,^22^ np‐Mo_2_C NWs,^29^ Co‐Mo_2_C,^30^ and MoC*_x_* nanooctahedrons^31^ (Table S5, Supporting Information). It is noticeable that the NRPGC electrode also shows remarkable HER activity (*U*_onset_ = 233 mV, η_10_ = 405 mV), outperforming most N‐doped graphene‐based substrate for HER in the literature.^20^, ^25^, ^32^ Anyway, the WN*_x_* and NRPGC electrodes show much worse performance than the WN*_x_*‐NRPGC electrode. It suggests a synergistic effect was likely created between WN*_x_* and NRPGC phases in the WN*_x_*‐NRPGC composites.

**Figure 3:**
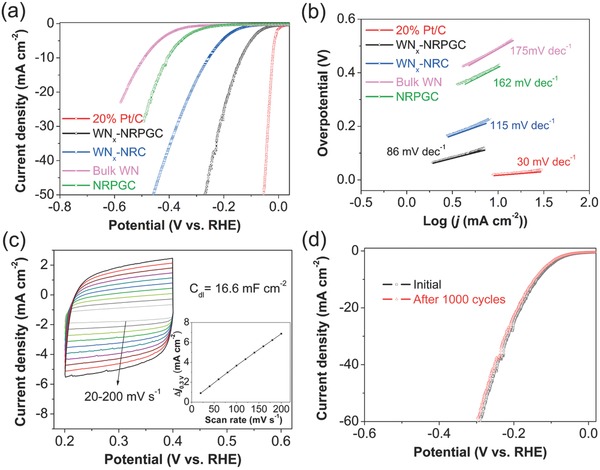
a) Polarization curves after iR compensation in 0.5 m H_2_SO_4_ at a scan rate of 5 mV s^−1^ and b) corresponding Tafel plots of WN*_x_*‐NRPGC, WN*_x_*‐NRC, NRPGC, Bulk WN, and 20% Pt/C catalysts. c) Electrochemical CV scans recorded for WN*_x_*‐NRPGC at different rates from 20 to 200 mV s^−1^ in the potential range of 0.2–0.4 V. Inset: Linear fitting of the capacitive currents versus CV scan rates for WN*_x_*‐NRPGC. d) Polarization curves of WN*_x_*‐NRPGC initially and after 1000 CV cycles.

It should be noted that better activity has always been achieved if a Pt wire or foil was used as the counter electrode. The HER activity of the WN*_x_*‐NRPGC catalyst determined using a Pt wire as the counter electrode (*U*_onset_ = 0 mV, η_10_ = 83 mV, Figure S8, Supporting Information) is also higher than that determined by a graphite rod as the counter electrode. It has been reported that Pt could be dissolved in acidic solution and further electrodeposit on the electrode material during long‐term operation, which will confuse the intrinsic HER performance of the tested catalyst.^33^ In this regard, graphite rod as the counter electrode is preferred.

To understand the detailed underlying mechanism of HER activity, Tafel plots were constructed from steady‐state polarization measurements. As shown in Figure 3b, the liner regions were fitted to Tafel equation (η = *b* log*j* + *a*, where *j* refers the current density and *b* refers the Tafel slope) to obtain slope, *b*. The Tafel slope of Pt/C is 30 mV dec^−1^, consistent with the reported values in literature.^25^ For comparison, the Tafel slope of the WN*_x_*‐NRPGC catalyst is 86 mV dec^−1^, which is lower than those of WN*_x_*‐NRC (115 mV dec^−1^), NRPGC (162 mV dec^−1^), and bulk WN (175 mV dec^−1^). It further suggests a synergistic effect was created between WN*_x_* and NRPGC phases in the WN*_x_*‐NRPGC sample, which led to a change in the reaction mechanism of HER. Generally, three principal steps noted as Volmer (120 mV dec^−1^), Heyrovsky (40 mV dec^−1^), and Tafel (30 mV dec^−1^) steps are involved in HER.^20^, ^25^ Based on the value of Tafel slope, the H_2_ production on the WN*_x_*‐NRPGC electrode occurs via the Volmer–Heyrovsky mechanism, where the rate‐determining step is the electrochemical desorption. Moreover, the exchanged current density (*j*_0_) reflects the intrinsic catalytic activity. By extrapolation to the Tafel plots (Figure S9, Supporting Information), the exchanged current density of the WN*_x_*‐NRPGC catalyst is 0.37 mA cm^−2^, nearly five times that of the WN*_x_*‐NRC (0.077 mA cm^−2^) and thirty times that of the bulk WN (0.013 mA cm^−2^, Table S5, Supporting Information), implying more favorable HER kinetics at the WN*_x_*‐NRPGC/electrolyte interface. Electrochemical impedance spectroscopy measurements were used to provide further insight into electrode kinetics of the obtained materials. The resulting Nyquist plot collected at η = 200 mV on the WN*_x_*‐NRPGC, WN*_x_*‐NRC, and Bulk WN electrodes are compared. As shown in Figure S10 of the Supporting Information, the charge‐transfer resistance (*R*_ct_) of the WN*_x_*‐NRPGC catalyst is much lower than that of the WN*_x_*‐NRC and Bulk WN catalysts. The lower *R*_ct_ is attributed to the highly porous graphene‐like carbon nanosheets of the WN*_x_*‐NRPGC, which allow rapid electron transfer during the electrochemical processes.

To further illustrate the superior HER performance of the WN*_x_*‐NRPGC electrode, the electrochemically active surface area (ECSA) was estimated from the corresponding electrochemical double‐layer capacitance (*C*_dl_), which is proportional to ECSA (Figure 3c).^25^ A voltammetric analysis of the WN*_x_*‐NRPGC electrode within the potential range of +0.2 to +0.4 V implies a *C*_dl_ value of 16.6 mF cm^−2^, which is much larger than those of the WN*_x_*‐NRC electrode (3.1 mF cm^−2^, Figure S11a,b, Supporting Information) and the bulk WN electrode (0.45 mF cm^−2^, Figure S11c,d, Supporting Information), but less than that of the NRPGC carbon (33.5 mF cm^−2^, Figure S11e,f, Supporting Information). The high *C*_dl_ value of the WN*_x_*‐NRPGC electrode suggests a higher ECSA, which was then determined on the basis of the capacitance measurement (see Supporting Information for detail). Figure S12 of the Supporting Information presents the polarization curves and the corresponding Tafel plots of the WN*_x_*‐NRPGC and WN*_x_*‐NRC electrodes normalized by ECSA.^34^, ^35^ It can be observed that the WN*_x_*‐NRPGC catalyst still exhibits better HER performance, lower Tafel slope, and larger *j*_0,ECSA_ than the WN*_x_*‐NRC electrode.

The electrocatalytic stability is an indispensable factor for practical catalytic application. The polarization curves before and after 1000 continuous cyclic voltammetric (CV) sweeps are recorded in Figure 3d. As seen, after cycling, the catalyst provides similar polarization curve to the initial cycle with negligible degradation in both the onset potential and the cathodic current density, confirming the remarkable stability of the catalyst in the acidic electrolyte. The composition characterization of the cycled WN*_x_*‐NRPGC electrode is shown in Figure S13 of the Supporting Information (with detailed illustration) and the corresponding TEM image (Figure S14, Supporting Information) reveals that the morphology as well as the crystallinity was well maintained after the cycling test. When further evaluated through prolonged electrolysis at a fixed potential of 180 mV, the WN*_x_*‐NRPGC electrode exhibited a stable current density at around 20 mA cm^−2^ for over 10 h in 0.5 m H_2_SO_4_ (Figure S15, Supporting Information), and only trace amount of W (Table S4, Supporting Information) was detected from the inductively coupled plasma (ICP) analysis of the electrolyte after the test, further confirming the durability of the WN*_x_*‐NRPGC material.

As demonstrated, the carbon derived from the pyrolysis of D201 resin under ammonia atmosphere and molten salt heating (NRPGC) had rich N content (9.5 at%). Both the quaternary amine of the resin and the NH_3_ atmosphere can act as the nitrogen sources for doping into the carbon structure. To give a deeper illustration, the N‐doped porous graphene‐like carbon (denoted as NPGC) was also synthesized through the same method as for NRPGC except that the ammonia atmosphere during the calcination process was replaced by argon. From the comparison of Raman spectra in **Figure** **4**a, the intensity ration of D band to G band increased from 1.15 for NPGC to 1.26 for NRPGC, suggesting more structure defects in NRPGC,^8^ presumably originating from the extra nitrogen doping from the NH_3_ treatment. Higher amount of lattice defect should benefit the HER.^[36]^ According to XPS, the total nitrogen contents are 2.3 at% and 9.5 at% for the NPGC and NRPGC samples, respectively (Figure S16, Supporting Information). It suggests a large portion of the doped nitrogen was originated from the calcination atmosphere of ammonia. Figure 4b shows the different types of nitrogen in NPGC and NRPGC. In comparison with the NPGC sample, the intensity of “pyridinic‐N” peak of NRPGC became more prominent, demonstrating that the pyrolysis under NH_3_ treatment mainly introduced pyridinic‐N into the carbon structure. As can be seen from Figure 4c, 54.3% of nitrogen in NRPGC sample took the form of pyridinic‐N, while the corresponding ratio in the NPGC is only 26.8%. Experimental results and DFT calculations have demonstrated that more nitrogen doping with high percentage of pyridinic N is effective in enhancing the HER activity.^36^

**Figure 4:**
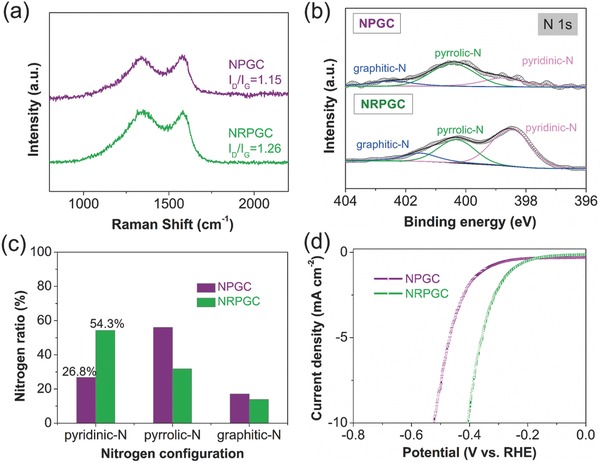
a) Raman spectra, b) high‐resolution N 1s spectra, c) comparison of the ratios of three doped N configurations and d) polarization curves of NRPGC and NPGC.

As expected, when evaluated under the electrochemical condition, the NRPGC electrode shows better HER performance than the NPGC electrode (Figure 4d), as indicated by the *U*_onset_ of 233 and 325 mV, η_10_ of 405 and 528 mV, Tafel slope of 162 and 185 mV dec^−1^ (Figure S17, Supporting Information), respectively. The larger *C*_dl_ of NRPGC (33.5 mF cm^−2^) than that of NPGC (21.2 mF cm^−2^, Figure S18, Supporting Information) also implies the larger available active sites of the N‐rich carbon. It was pointed out that an interaction between C‐pyridinic N and the composited metal phase could appear to introduce a synergistic effect,^7^, ^20^ which may account for the appearance of synergistic effect between WN*_x_* and NRPGC in the WN*_x_*‐NRPGC composite. On this basis, during the HER process, the pyridinic N‐enriched NC substrate of the WN*_x_*‐NRPGC composite not only provides more H* active adsorption sites, but also reinforces the synergistic effect between the C‐pyridinic N and the WN*_x_* phase, thus contributing to a superior activity.

Based on above analysis, the enhanced HER catalytic activity of the WN*_x_*‐NRPGC composite can be attributed to the following aspects. First, resulting from the atom level distribution of WO_4_^2−^ over the resin structure from the ion exchange, the growth of WN*_x_* nanoparticles was suppressed during the pyrolysis process, thus creating more available active sites for HER. Second, the graphene‐like carbon nanosheets with robust adhesion of WN*_x_* nanoparticles can maintain the structure stability and endow with rapid electron transfer from WN*_x_* to the electrode. Third, the highly porous structure is expected to maximize the number of exposed active sites and facilitate charge and mass transfer during the electrochemical reactions. Fourth, the abundant nitrogen doping with high percentage of pyridinic‐N in the carbon support can improve the conductivity of the carbon and increase the H* adsorption site, meanwhile, the increased pyridinic N doped configuration together with the increased synergistic effect between the C‐pyridinic N and the WN*_x_* phase contribute to a better HER performance. At last, the robust conjugation of carbon support and WN*_x_* phase guarantees a strong corrosion resistance to acid, confirming the stability during long‐term operation.

In summary, a new hybrid of WN*_x_*‐NRPGC has been synthesized using a combined ion‐exchange/molten salt strategy under ammonia atmosphere. The nanosized WN*_x_* particles, the conducted graphene‐like carbon networks, especially the enriched nitrogen doping with high percentage of pyridinic‐N contribute to the prominent electrocatalytic activity. The fabrication is facile and scalable, and more importantly, this method can potentially be applied to prepare other nonprecious metal‐nitrogen rich graphene‐based electrocatalyst for HER.

## Experimental Section

Experimental details are included in the Supporting Information.

## Conflict of Interest

The authors declare no conflict of interest.

## Supporting information

Supplementary
