# Supplementary material for: Rationally Designed Hierarchically Structured Tungsten Nitride and Nitrogen‐Rich Graphene‐Like Carbon Nanocomposite as Efficient Hydrogen Evolution Electrocatalyst
Source: Adv Sci (Weinh). 2017 Dec 8;5(2):1700603. doi: 10.1002/advs.201700603 (PMC5827495; doi:10.1002/advs.201700603)
Supplement: Supplementary file 1 — Supplementary [file ADVS-5-1700603-s001.pdf]

Copyright WILEY-VCH Verlag GmbH & Co. KGaA, 69469 Weinheim, Germany, 2017.

ADVANCED SCIENCE
Open Access

Supporting Information

for *Adv. Sci.,* DOI: 10.1002/advs.201700603

Rationally Designed Hierarchically Structured Tungsten Nitride and Nitrogen-Rich Graphene-Like Carbon Nanocomposite as Efficient Hydrogen Evolution Electrocatalyst

*Yanping Zhu, Gao Chen, Yijun Zhong, Wei Zhou, and Zongping Shao**

Copyright WILEY-VCH Verlag GmbH & Co. KGaA, 69469 Weinheim, Germany, 2016.

Supporting Information

Rationally designed hierarchically structured tungsten nitride and nitrogen-rich graphene-like carbon nanocomposite as efficient hydrogen evolution electrocatalyst

*Yanping Zhu, Gao Chen, Yijun Zhong, Wei Zhou and Zongping Shao**

Y. Zhu, G. Chen, Prof. W. Zhou, Prof. Z. P. Shao
Jiangsu National Synergetic Innovation Center for Advanced Materials (SICAM), State Key Laboratory of Materials-Oriented Chemical Engineering, College of Chemical Engineering, Nanjing Tech University, No.5 Xin Mofan Road, Nanjing 210009, P.R. China
Y. Zhong, Prof. Z. P. Shao
Department of Chemical Engineering, Curtin University, Perth, Western Australia 6845, Australia
Prof. Z. P. Shao
College of Energy, Nanjing Tech University, No.5 Xin Mofan Road, Nanjing 210009, P.R. China

E-mail: shaozp@njtech.edu.cn

I. Experimental Section

Materials Preparation

WN_x-NRPGC. All the chemicals employed in this work were analytical regents without further treatment. Typically, 5 g of the pretreated macroporous cinnamic anion-exchange resin (D201) was impregnated with target ions of tungsten in a 0.3 M sodium tungstate dihydrate ($Na_2WO_4 \cdot 2H_2O$) aqueous solution (50 mL) and stirred magnetically for 6 h at room temperature (RT). The exchanged resin was collected after rinsing, drying and grinding. Then 1 g of the adsorbed resin powder was added into 100 mL of molten salt (MS) mixture-aqueous solution containing 5 g of potassium chloride (KCl) and 5 g of sodium chloride (NaCl) under vigorously stirring at 80 ^oC in oil bath to allow the water to evaporate slowly. Finally, the solid resin-eutectic salt mixture was transferred to a quartz boat and calcined at 800 ^oC for 2 h (ramp rate: 2 ^oC min^{-1}) under an NH_3 flow. After cooling naturally to RT, the product was introduced into the distilled water and stirred for several hours to remove the residual salt porogen. Afterwards, the sample was subsequently obtained by repeatedly washing with deionized water and vacuum desiccated at 60 ^oC for 12 h. A fine black powder was finally obtained.

In control experiments, samples with different concentrations of the W precursor (0.1 M, 0.2 M, 0.4 M) were prepared under the same condition. To investigate the effect of nitridation temperature on the electrocatalytic performance, the nitridation was also carried out at 700 ^oC and 900 ^oC while keeping other parameters unchanged.

WN_x-NRC. This reference sample was fabricated following the same procedure as for WN_x-NRPGC without the attendance of MS.

Bulk WN. This reference sample was prepared by the calcination of bulk WO_3 (99.9%, Sinopharm Chemical Reagent Co., Ltd.) at 800 ^oC for 2 h under an ammonia atmosphere.

NRPGC. This reference sample was obtained following the same procedure as for WN_x-NRPGC without the presence of $Na_2WO_4 \cdot 2H_2O$.

NPGC. This reference sample was synthesized through the same method as for NRPGC except that the ammonia atmosphere during the calcination process was replaced by argon.

Commercial Pt/C (20 wt%, Johnson-Matthey) was purchased and used as received.

Materials characterization:

The crystallographic information of the samples was evaluated through RT powder X-ray diffraction (XRD, Rigaku Smartlab, Cu kα radiation) measurement over a 2θ range from 10° to 90°. The microstructure images were observed with field-emission scanning electron microscope (FE-SEM) (HITACHI S-4800), transmission electron microscope (TEM) and high-resolution transmission electron microscope (HR-TEM) (JEOL JEM-200CX). X-ray photoelectron spectroscopy (XPS) analysis was carried out using a PHI550 system and the as-obtained spectra were fitted through XPSPEAK41 software. Raman spectra were collected by an HR800 UV micro-Raman spectrometer. Nitrogen adsorption-desorption isotherms were measured using an adsorption-desorption instrument (BELSORP-MINI, Japan) with nitrogen at 77 K. The samples were pretreated at 200 °C for 2 h under vacuum to remove surface-adsorbed species before analysis. Specific surface area of the samples was calculated using the Brunauer-Emmett-Teller (BET) equation.

Electrochemical measurements:

Electrochemical measurements were conducted at room temperature in a standard three-electrode electrochemical cell with a CHI 760E bipotentiostat. All tests were performed in a 0.5 M H_2SO_4 aqueous solution. An Ag/AgCl electrode (3 M KCl) and graphite rod were used as the reference and counter electrode, respectively. 5 mg of the measured material (WN_x-NRPGC, WN_x-NRC, Bulk WN, NRPGC, NPGC, 20% Pt/C) and 50 uL 5 wt% Nafion solution were dispersed in 0.5 mL water/ethanol mixture (v_w/v_e=4:1) by mild sonication. Then, a 5-μL aliquot of the homogeneous catalyst ink was pipetted onto the surface of the GC substrate (0.1256 cm^2), yielding an approximate catalyst loading of 0.362 mg cm^{-2}. The as-prepared catalyst film was dried at room temperature.

Hundreds of CV cycles were performed until the CV curves remained unchanged, suggesting that the surface metal oxides and residue organics and ions have been removed. All currents presented in this work were normalized with respect to the geometrical surface area of GC. All the potentials shown were calibrated with respect to the reversible hydrogen electrode (RHE) after IR correction using the following equation: E(RHE) = E(Ag/AgCl) + 0.2374 V. Thousands of potential cycles were conducted with continuous argon gas purging in the potential region from 0 to -0.8 V (vs Ag/AgCl) at a sweep rate of 100 mV s^{-1} under 2400 rpm till the cyclic voltammetry curves (CVs) stay unchanged. Polarization curves were recorded using linear sweep voltammetry (LSV) from 0 to -0.6 V (vs Ag/AgCl) at a sweep rate of 5 mV s^{-1} under 2400 rpm. The polarization curves were replotted as overpotential (η) versus the logarithm of current density (log $|j|$) to obtain Tafel plots. The linear portions of the Tafel plots were then fitted to the Tafel equation ($\eta = b \log |j| + a$) to obtain the Tafel slope (b). The cyclic voltammograms(CVs) obtained with different rates from 20 to 200 mV s^{-1} in the potential range of 0.2-0.4 V (vs. RHE) were recorded to calculate the electrochemical double-layer capacitances (EDLCs), $C_{dl.}$ The accelerated stability test was studied at room temperature by potential cycling between 0 and -0.6 V (vs Ag/AgCl) at a sweep rate of 100 mV s^{-1} for a given number of cycles. The long-term stability was also tested under a static overpotential of 180 mV for 10 hours. The electrochemical impedance spectroscopy (EIS) measurements were carried out at an overpotential of 200 mV under the influence of an ac voltage of 5 mV from 10^5 to 0.1 Hz.

II. Supplementary Results

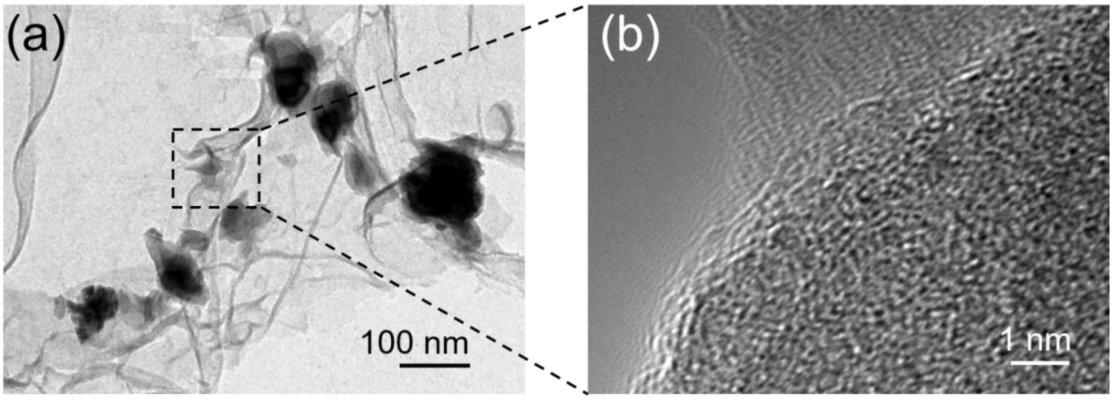

Figure S1. (a) TEM and (b) HRTEM images of WN_x-NRPGC

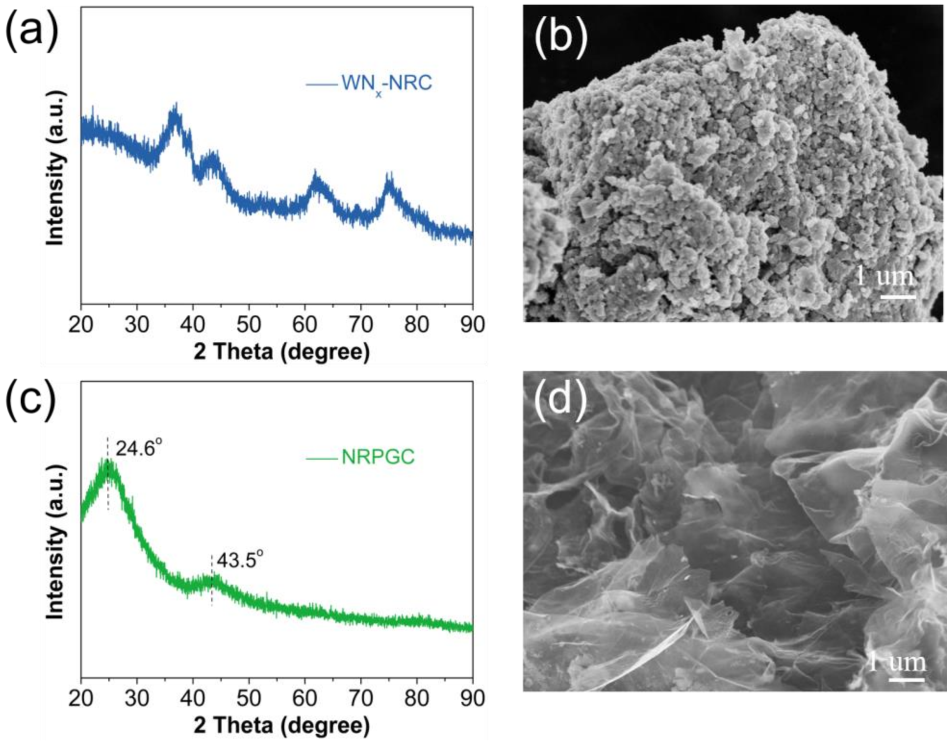

Figure S2. XRD patterns and the corresoponding SEM images of (a,b) WN_x-NRC and (c,d) NRPGC.

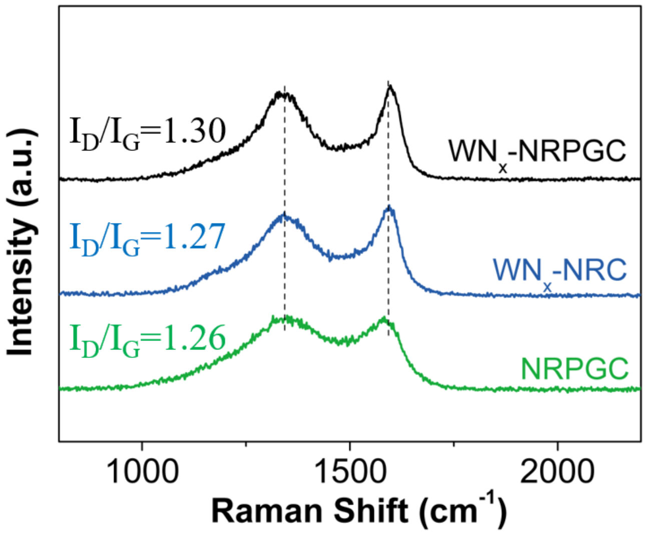

Figure S3 Raman spectra of WN_x-NRPGC, WN_x-NRC and NRPGC.

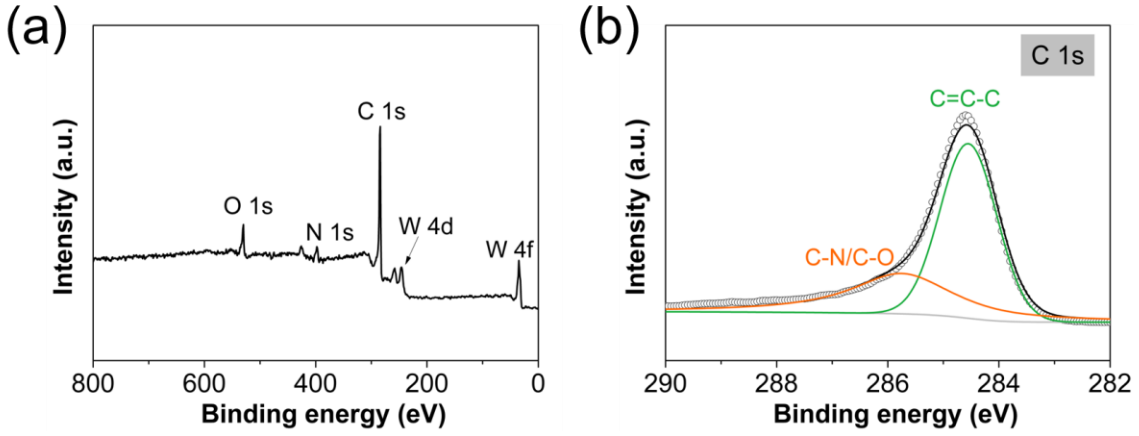

Figure S4 (a) Survey XPS spectrum and (b) High-resolution spectra of C 1s of WN_x-NRPGC.

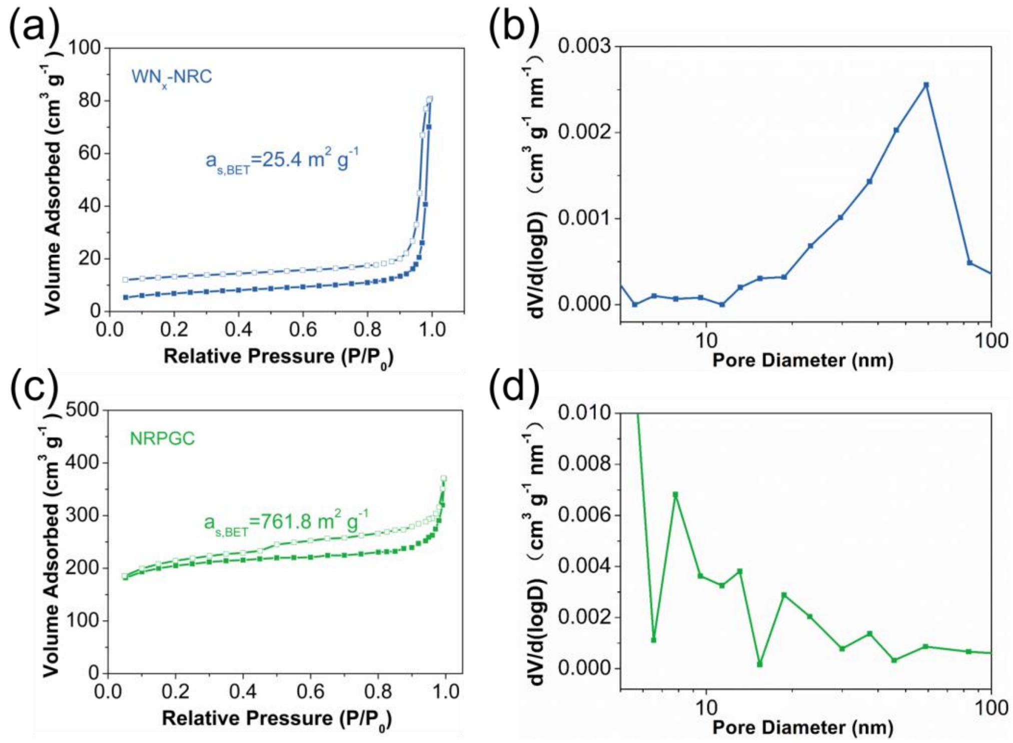

Figure S5. N_2 adsorption-desorption isotherms and the corresponding pore size distribution curves of (a,b) WN_x-NRC and (c,d) NRPGC.

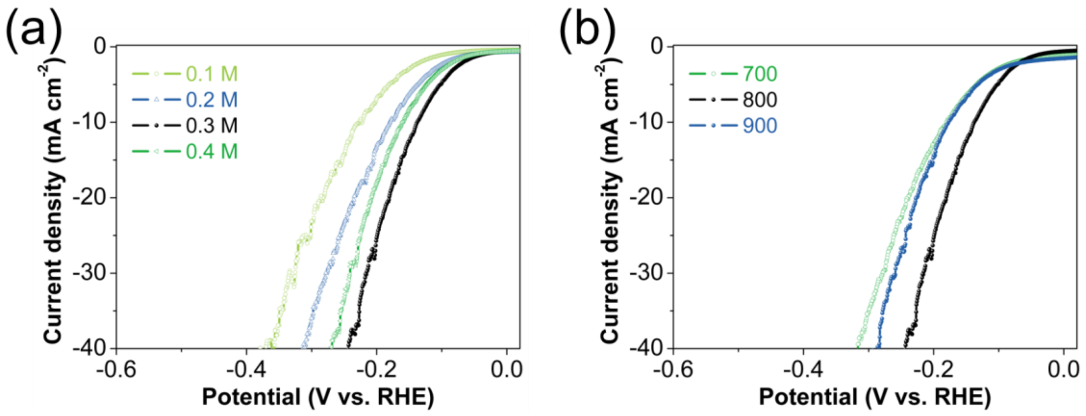

Figure S6. Comparisons of catalytic activities of samples (a) from different amount of W source and (b) annealed at different temperature in 0.5 M H_2SO_4

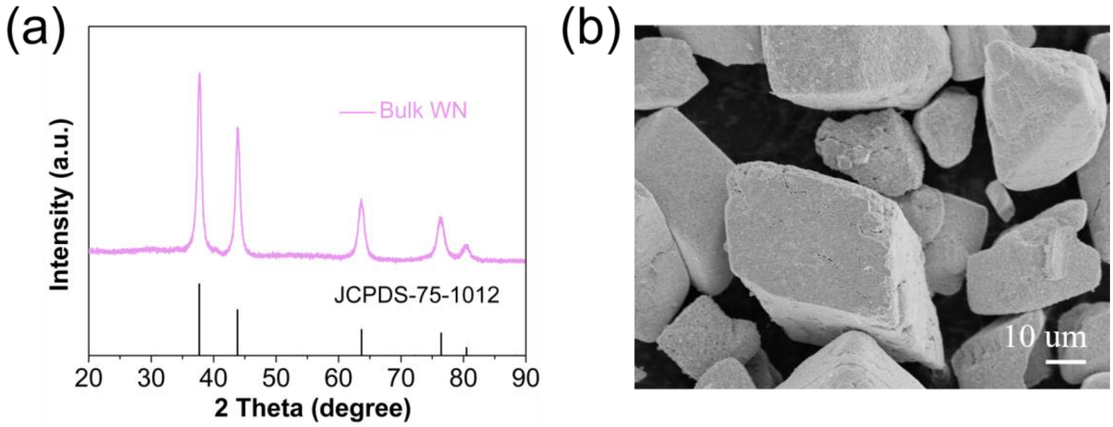

Figure S7. (a) XRD patterns and (b) the corresoponding SEM image of the bulk WN.

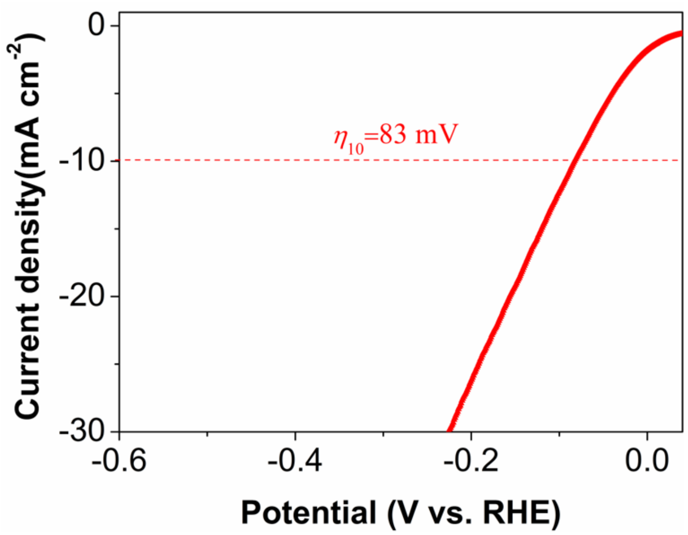

Figure S8. Polarization curve of WN_x-NRPGC with Pt wire as counter electrode.

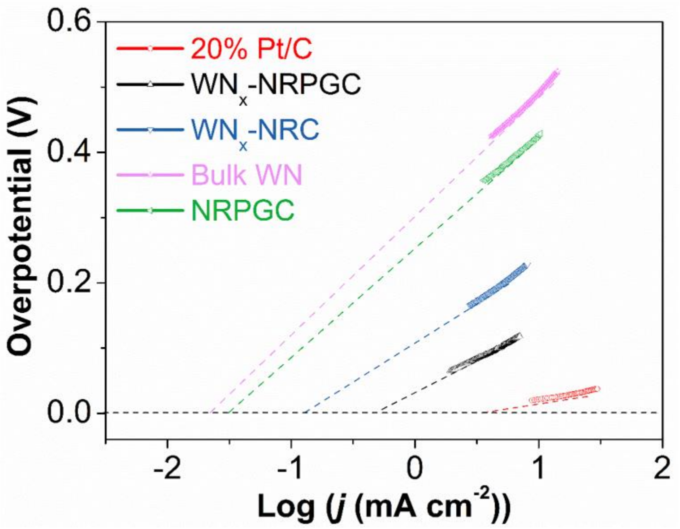

Figure S9. Calculated exchange current densities of the five samples by applying extrapolation method to the Tafel plots.

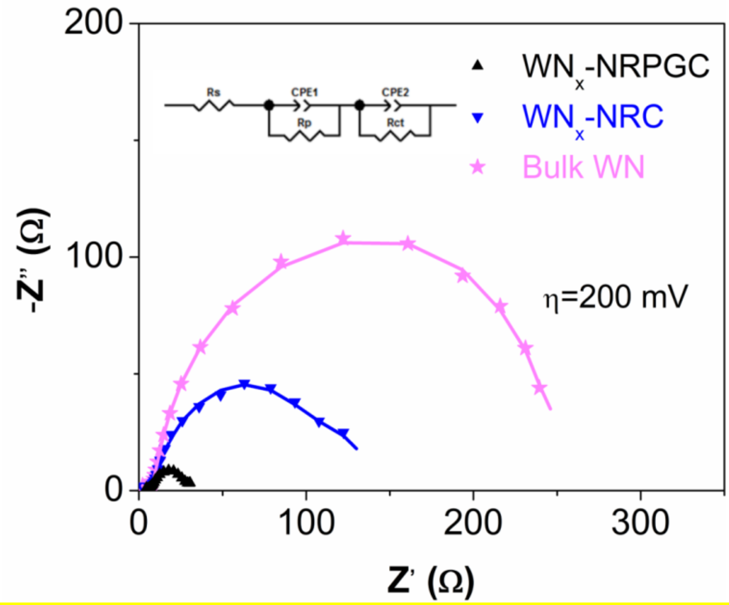

Figure S10. EIS Nyquist plots of WN_x-NRPGC, WN_x-NRC and Bulk WN catalysts collected at an HER overpotential of 200 mV.

In the equivalent circuit model, the R_s, $R_{p,}$ and R_{ct} in the equivalent circuit represent the resistance of the electrolyte, the resistance to mass transfer through the porous electrode, and the resistance to charge transfer, respectively. The CPE is an constant phase element that is used to approximate the electrode processes in the real-word situation, which cannot be adequately described by simple capacitors and resistors. The electro-catalytic activity is directly related to the resistance to charge transfer, R_{ct}.

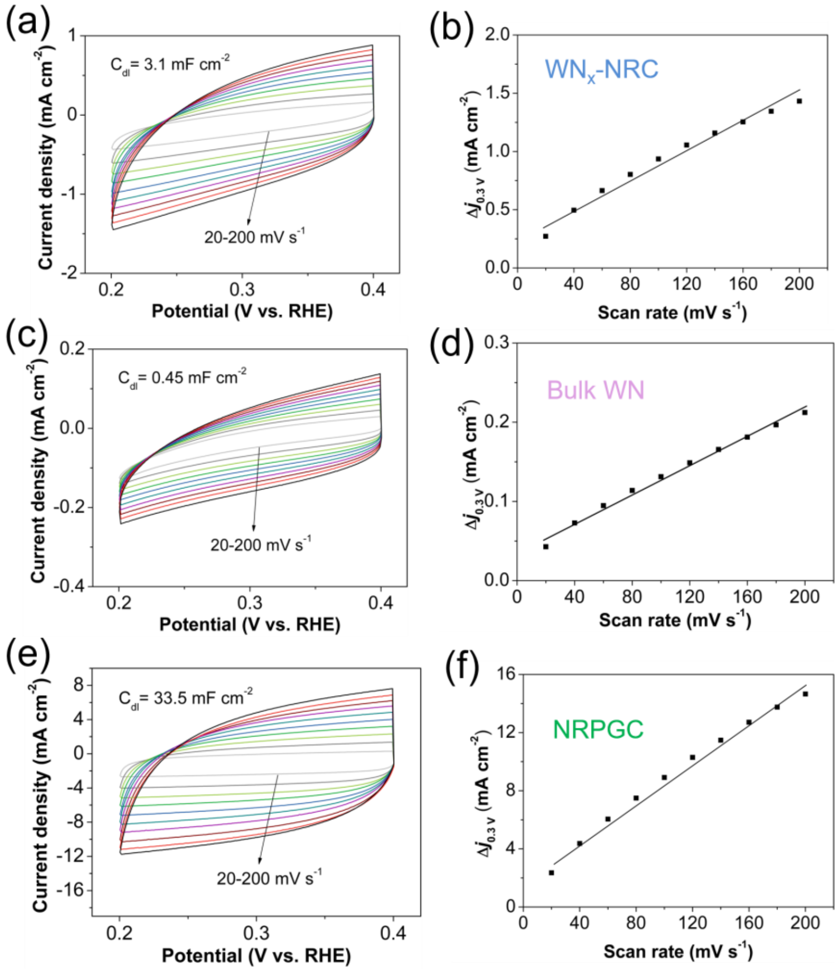

Figure S11. Electrochemical CV scans and the the corresponding linear fitting of the capacitive currents versus CV scan rates recorded for (a,b) WN_x-NRC, (c,d) Bulk WN and (e,f) NRPGC.

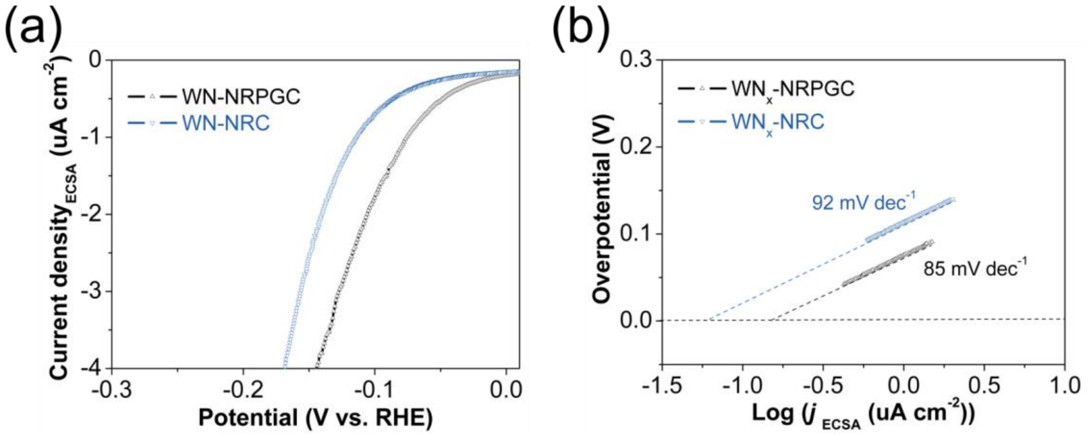

Figure S12. (a) Polarization curves and (b) Tafel plots normalized by ECSA for WN_x-NRPGC and WN_x-NRC recorded in 0.5 M H_2SO_4.

ECSA calculation:

The electrochemical active surface area (ECSA) can be estimated using the capacitance (C). The specific capacitance for a flat surface is generally found to be in the range of 20~60 μF cm^{-2}. 40 μF cm^{-2} was used in the following calculations of the ECSA as literatures generally did [1,2].

The following formula was used to calculate ECSA:

$$\mathrm{ECSA} = \frac{\mathrm{C}}{40\ \mathrm{uF\ cm^{-2}\ per\ cm^{-2}}}$$

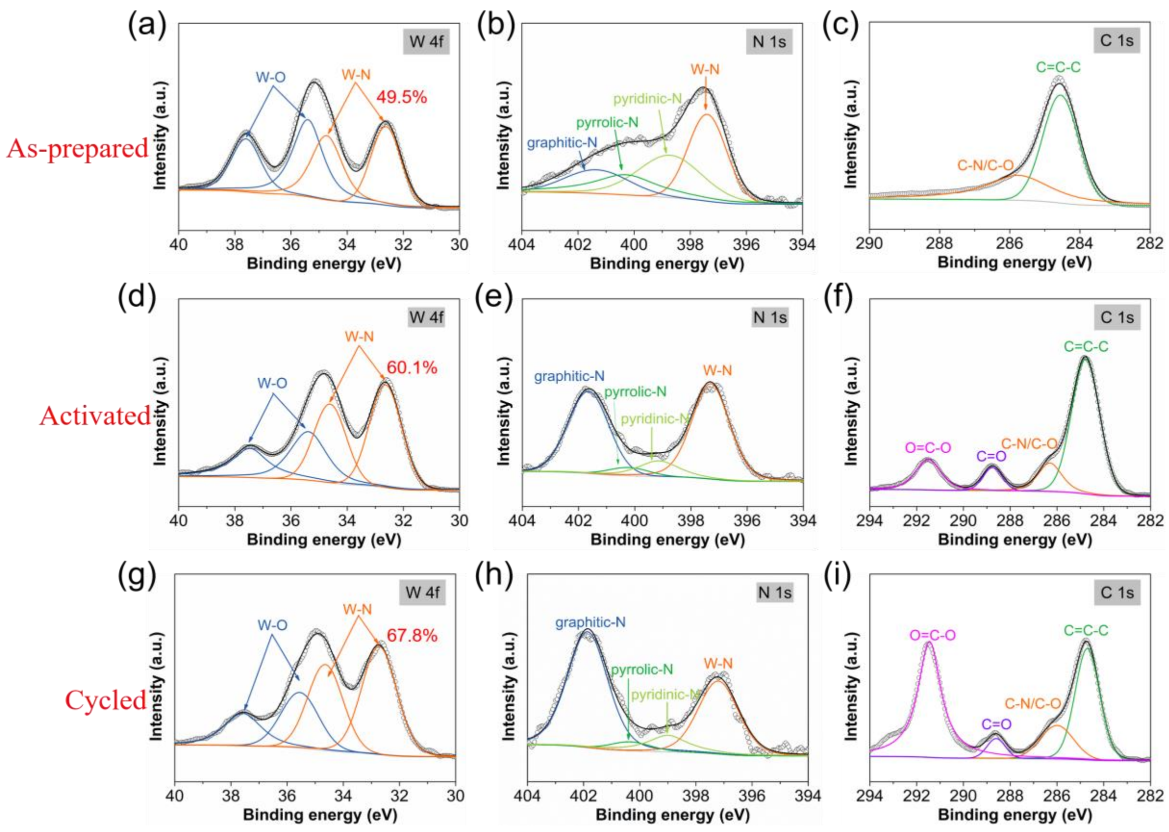

Figure S13. High-resolution XPS spectra of W4f, N 1s and C 1s (a, b, c) before activation, (d, e, f) after activation and (g, h, i) after cycling.

Shown in **Figure S13** is the XPS analysis of the activated WN_x-NRPGC and the cycled WN_x-NRPGC. After the activation, in comparison with the as-prepared sample, the intensity of the W-O peaks (**Figure S13d**) became less prominent, indicating partial removal of the surface metal oxides during the repeated CV scans. From **Figure S13e**, the intensity of graphitic-N increased in the track of the N 1s electron. New oxidized forms of carbon were observed and ascribed to an epoxy carbon and a carboxyl group, respectively (**Figure S13f**). It can be intuitively speculated that, after the activation process, the increased exposure of WN (resulted from the partial removal of the surface metal oxides) as well as the growth of negatively charged carboxyl/epoxy group on the surface of carbon enhanced the bonding to protons and weakened the negative effects of the reduced ratio of pyridinic-N, leading to an improved HER activity **[3]**.

When a steady performance was reached after number of CV cycles, the polarization curve of the WN_x-NRPGC catalyst after 1000 continuous CV sweeps were recorded. Compared with the activated sample, the cycled catalyst has similar composition except the increase exposure of WN (**Figure S13g**, 67.8%, positive effect), the increase of graphitic-N (**Figure S13h**, negative effect) and the increase of carbon oxide species (**Figure S13i**, positive effect). The three effects neutralized, leading to a steady HER performance.

As for the increase of graphitic-N during the activation process, deeper research on the change of N doped configuration during the HER process by *in-situ* characterization techniques and theoretical calculations are encouraged.

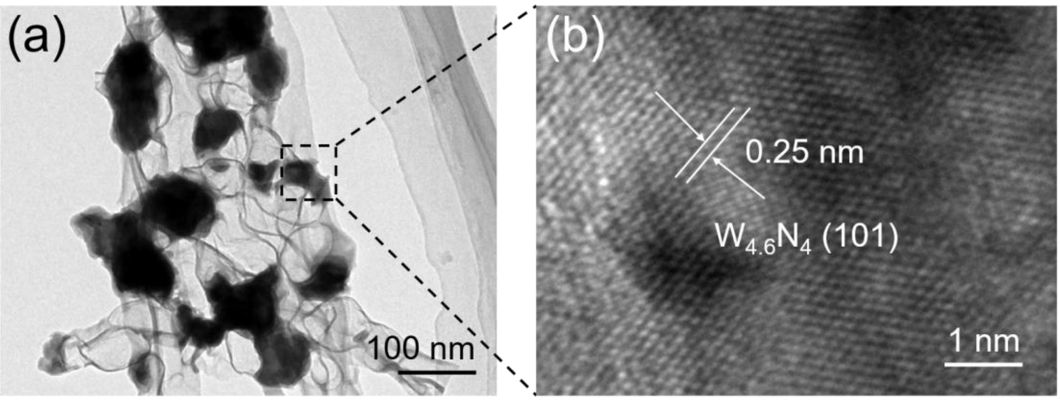

Figure S14. TEM and HRTEM images of the WN_x-NRPGC after electrochemical performance test.

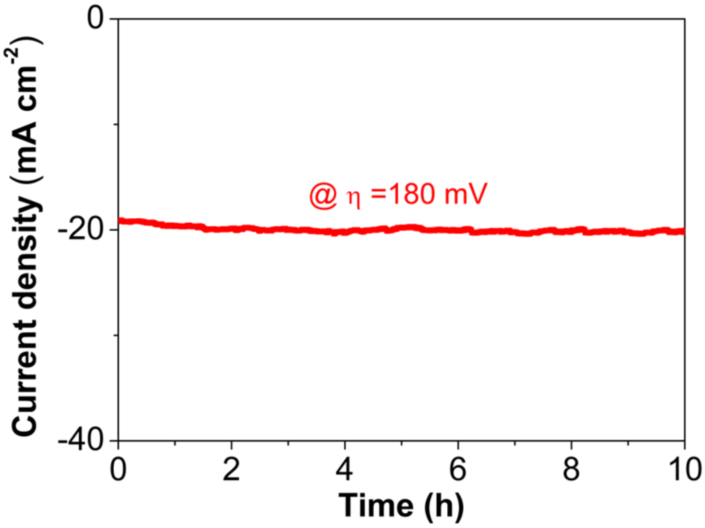

Figure S15. Time-dependent current density curve of WN_x-NRPGC under a static overpotential of 180 mV for 10 h.

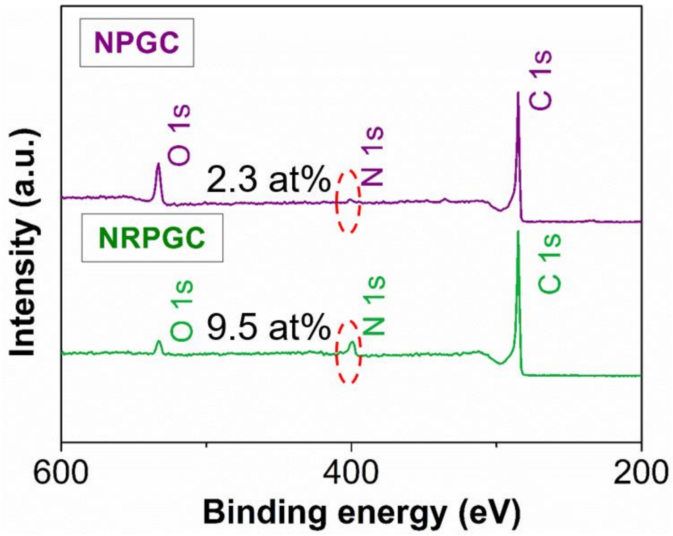

Figure S16. Survey XPS spectra of NPGC and NRPGC.

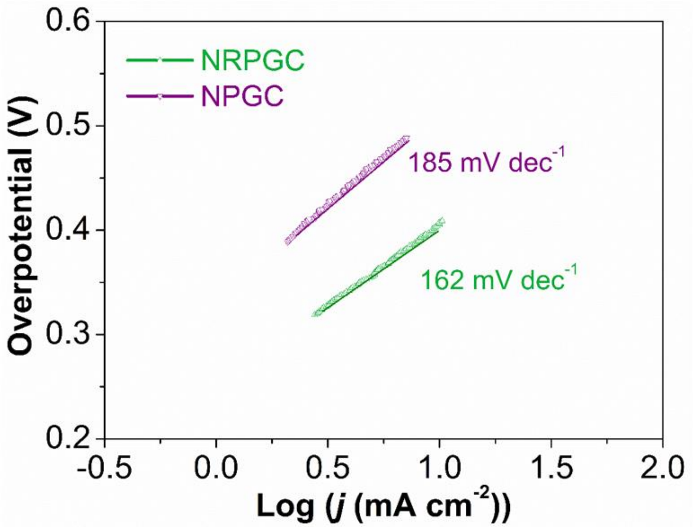

Figure S17. Tafel plots of NRPGC and NPGC catalysts.

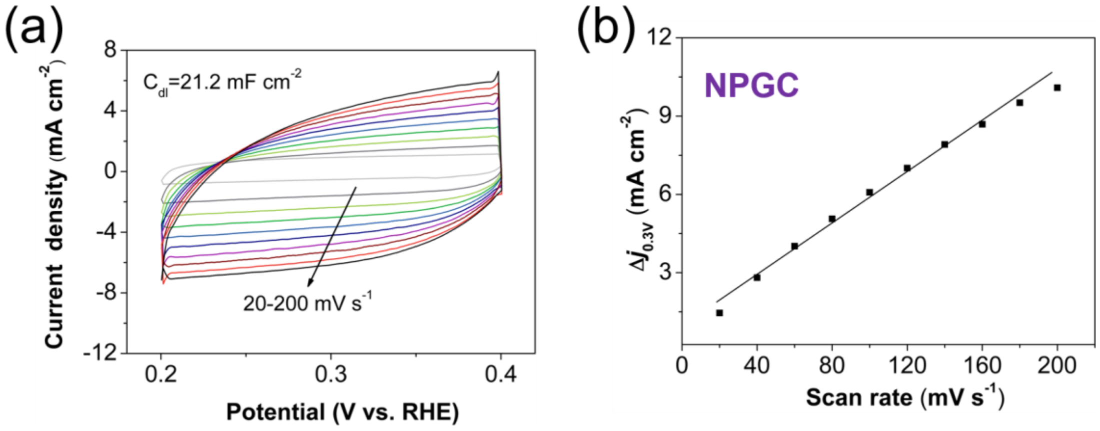

Figure S18. Electrochemical CV scans recorded for NPGC at different rates from 20 to 200 mV s^{-1} in the potential range of 0.2-0.4 V, (b) Linear fitting of the capacitive currents versus CV scan rates.

Table S1 Physical properties of the various materials.

Sample	$a_{s,BET}$ $m^2\ g^{-1}$	$V_{Total}(P/P_0 = 0.99)$ $cm^3\ g^{-1}$
WN_x-NRPGC	**304**	**0.35**
WN_x-NRC	25.4	0.11
NRPGC	761.8	0.39
Bulk WN	19.8	0.072

Table S2 Comparison of catalytic parameters of different HER catalysts.

Catalyst	Onset potential (mV vs RHE)	Overpotential at 10 mA cm^{-2} (mV vs RHE)	j_0 (mA cm^{-2})	Tafel slope (mV dec^{-1})
20% Pt/C	0	22	1.84	30
WN_x-NRPGC	**30**	**132**	**0.347**	**86**
WN_x-NRC	123	255	0.077	115
NRPGC	233	405	0.027	162
Bulk WN	331	492	0.013	175

Table S3 Comparison of the Capacitance (C), ECSA, Tafel Slope, and $j_{0,ECSA}$ of WN_x-NRPGC and WN_x-NRC.

Catalyst	C (mF cm^{-2})	ECSA (cm^2)	Tafel slope (mV dec^{-1})	$j_{0,ECSA}$ (uA cm^{-2})
WN_x-NRPGC	**16.6**	**415**	**85**	**0.13**
WN_x-NRC	3.1	77.5	92	0.06

Table S4 ICP analysis of the electrolyte before and after long durability test.

Sample	W (ppm)
Before	0
After	2.3

Table S5 Comparison of electrocatalytic HER activity in acidic conditions (0.5 M H_2SO_4) for WN_x-NRPGC with some of the most active non-precious HER catalysts ever reported.

Catalyst	Loading (mg cm^{-2})	η_{10} (mV)	Tafel slope (mV dec^{-1})	Reference
WN_x-NRPGC	**0.362**	**132**	**86**	**This work**
*WN_x-NRPGC	0.362	83	79	**This work**
W_2C/MWNT	0.556	123	45	[4]
P-Mo_2C@C NWs	1.3	89	42	[5]
np-Mo_2C NWs	0.21	135	60	[6]
Mo_xC nano-octahedrons	0.8	142	53	[7]
MoC-Mo_2C-31.4	0.14	126	43	[8]
Co-Mo_2C-0.02	0.14	140	39	[9]
Mo_2C@NC	0.28	124	60	[10]
*Mo_2C@NPC/NPRGO	0.14	34	33.6	[11]
*Mo_xC-Ni@NCV	1.1	68	45	[12]
*MoDCA-5	0.25	78	41	[13]
*Mo_2C/CNT-graphene	0.65	130	58	[14]
MoP|S	0.17	64	50	[15]
*P-WN/rGO	0.337	85	54	[16]
*MoCN NPs	0.4	140	46	[17]
Metallic WO_2-carbon	0.35	58	46	[18]
*β-Mo_2C/rGO	0.47	109	66.4	[19]
MoP	0.39	150	54	[20]
WS_2 nanosheets	1	142	70	[21]
Co_2P nanorods	1.02	134	51.7	[22]
Ni_2P nanoparticles	1	100	46	[23]

*the HER performance with Pt foil or wire as the counter electrode

References

[1] X. D. Wang, Y. F. Xu, H. S. Rao, W. J. Xu, H. Y. Chen, W. X. Zhang, D. B. Kuang, C. Y. Su, *Evolution. Energy Environ. Sci.*, **2016**, *9*, 1468.

[2] Y. Y. Chen, Y. Zhang, W. J. Jiang, X. Zhang, Z. H. Dai, L. J. Wan, J. S. Hu, *ACS Nano*, **2016**, *10*, 8851.

[3] Y. Zhu, G. Chen, X. Xu, G. Yang, M. Liu, *ACS Catal.*, **2017**, *7*, 3540.

[4] Q. F. Gong, Y. Wang, Q. Hu, J. G. Zhou, R. F. Feng, P. N. Duchesne, P. Zhang, F. J. Chen, N. Han, Y. F. Li, C. H. Jin, Y. G. Li, S.-T. Lee, *Nat. Commun.*, **2016**, *7*.

[5] Z. P. Shi, K. Q. Nie, Z. J. Shao, B. X. Gao, H. L. Lin, H. B. Zhang, B. L. Liu, Y. X. Wang, Y. H. Zhang, X. H. Sun, X. M. Cao, P. Hu, Q. S. Gao, Y. Tang, *Energy Enriron. Sci.*, **2017**, *10*, 1262.

[6] L. Liao, S. N. Wang, J. J. Xiao, X. J. Bian, Y. H. Zhang, M. D. Scanlon, X. L. Hu, Y. Tang, B. H. Liu, H. H. Girault, *Energy Environ. Sci.*, **2014**, *7*, 387.

[7] H. B. Wu, B. Y. Xia, L. Yu, X. Y. Yu, X. W. Lou, *Nat. Commun.*, **2015**, *6*, 6512.

[8] H. L. Lin, Z. P. Shi, S. N. He, X. Yu , S. N. Wang, Q. S. Gao, Y. Tang, *Chem. Sci.*, **2016**, *7*, 3399.

[9] H. L. Lin, N. Liu, Z. P. Shi, Y. L. Guo, Y. Tang, Q. S. Gao, *Adv. Funct. Mater.*, **2016**, *26*, 5590.

[10] Y. P. Liu, G. T. Yu, G. D. Li, Y. H. Sun, T. Asefa, W. Chen, X. X. Zou, *Angew. Chem. Int. Ed.*, **2015**, *54*, 10752.

[11] J. S. Li, Y. Wang, C. H. Liu, S. L. Li, Y. G. Wang, L. Z. Dong, Z. H. Dai, Y. F. Li, Y. Q. Lan, *Nat. Commun.*, **2016**, *7*.

[12] S. P. Wang, J. Wang, M. L. Zhu, X. B. Bao, B. Y. Xiao, D. F. Su, H. R. Li, Y. Wang, *J. Am. Chem. Soc.*, **2015**, *137*, 15753.

[13] R. G. Ma, Y. Zhou, Y. F. Chen, P. X. Li, Q. Liu, J. C. Wang, *Angew. Chem. Int. Ed.*, **2015**, *54*, 14723.

[14] D. H. Youn, S. Han, J. Y. Kim, J. Kim, Y. H. Park, S. H. Choi, J. S. Lee, *ACS Nano*, **2014**, *8*, 5164.

[15] J. Kibsgaard, T. F. Jaramillo, *Angew. Chem. Int. Ed*, **2014**, *53*, 14433.

[16] H. J. Yan, C. G. Tian, L. Wang, A. P. Wu, M. C. Meng, L. Zhao, H. G. Fu, *Angew. Chem. Int. Ed.*, **2015**, *54*, 6325.

[17] Y. Zhao, K. Kamiya, K. Hashimoto, S. Nakanishi, *J. Am. Chem. Soc.*, **2015**, *137*, 110.

[18] R. Wu, J. F. Zhang, Y. M. Shi, D. L. Liu, B. Zhang, *J. Am. Chem. Soc.*, **2015**, *137*, 6983.

[19] W. F. Chen, S. Iyer, S. Iyer, K. Sasaki, C. H. Wang, Y. M. Zhu, J. T. Muckerman, E. Fujita, *Energy Environ. Sci.*, **2013**, *6*, 1818.

[20] Z. C. Xing, Q. Liu, A. M. Asiri, X. P. Sun, *Adv. Mater.*, **2014**, *26*, 5702.

[21] M. A. Lukowski, A. S. Daniel, C. R. English, F. Meng, A. Forticaux, R. J. Hamers, S. Jin, *Energy Environ. Sci.*, **2014**, *7*, 2608.

[22] Z. P. Huang, Z. Z. Chen, Z. B. Chen, C. C. Lv, M. G. Humphrey, C. Zhang, *Nano Energy*, **2014**, *9*, 373.

[23] E. J. Popczun, J. R. McKone, C. G. Read, A. J. Biacchi, A. M. Wiltrout, N. S. Lewis, R. E. Schaak, *J. Am. Chem. Soc.*,**2013**, *135*, 9267.
